# Fast-track pathway for giant cell arteritis: Improved visual outcomes and reduced healthcare costs

**DOI:** 10.1371/journal.pone.0338397

**Published:** 2025-12-04

**Authors:** Loïc Duron, Thibaud Chazal, Thomas Sene, Julien Savatovsky, Augustin Lecler

**Affiliations:** 1 Department of Neuroradiology, Foundation Adolphe de Rothschild Hospital, Paris, France; 2 Université Paris Cité, Faculté de Médecine, Paris, France; 3 Department of Internal Medicine, Foundation Adolphe de Rothschild Hospital, Paris, France; University of Delhi, INDIA

## Abstract

**Background:**

Giant cell arteritis (GCA) is the leading vasculitis threatening vision in adults aged ≥ 50 years; permanent vision loss may occur within the first few days after symptom onset. We assessed the impact of a fast-track pathway (FTP) for early diagnosis and treatment of giant cell arteritis in terms of hospitalization patterns and cost-effectiveness.

**Methods:**

We conducted a retrospective, single-center medico-economic study of consecutive patients referred to a neuro-ophthalmology tertiary center between Nov 1, 2016, and Dec 31, 2022. GCA was defined by ≥ 3 American College of Rheumatology criteria plus a positive temporal-artery biopsy or vascular imaging. An FTP—24/7 access to internal medicine specialists, priority magnetic-resonance imaging, and protocol-driven corticosteroid initiation—was launched on Nov 1, 2018. Demographic, clinical, biological, care-pathway, and cost data were compared before (pre-FTP) and after (post-FTP) implementation. Continuous variables were analyzed with two-sample t tests or Wilcoxon rank–sum tests; categorical variables with χ² or Fisher’s exact test.

**Findings:**

We included 135 patients (mean age 76 ± 8 years, 61% women): 23 pre-FTP and 112 post-FTP. Baseline characteristics were similar between groups. Compared with the pre-FTP period, the FTP reduced full hospitalizations (62% [69/112] vs 96% [22/23]; p < 0.01) and increased day-hospital or outpatient management (39% vs 4%; p < 0.01). More patients received treatment within one month of symptom onset (54% vs 22%; p < 0.01). Final visual acuity improved (median 2.0 vs 2.6 logMAR; p < 0.01), while cumulative intravenous corticosteroid exposure was significantly reduced (1679 ± 760 mg vs 2295 ± 1055 mg; p = 0.02). Reliance on temporal-artery biopsy fell (17% vs 91%; p < 0.01), owing to a four-fold rise in diagnostic MRI use. Mean total medical costs decreased by €814 per patient (€3672 ± 2861 vs €4486 ± 3193), although this difference did not reach statistical significance (p = 0.23).

**Interpretation:**

A dedicated fast-track pathway for suspected GCA enables prompt, largely ambulatory care, halves unnecessary full hospitalizations, speeds treatment initiation, improves visual prognosis, and lowers overall expenditure. These findings support wider adoption of imaging-driven FTPs to mitigate the growing clinical and economic burden of GCA.

## Introduction

Giant Cell Arteritis (GCA) is the most prevalent chronic form of vasculitis affecting medium and large arteries in people older than 50 years [1]. Pathological changes associated with GCA include arterial wall inflammation and thickening that can lead to ischemic complications, with reported visual manifestations in about 30% of GCA patients and permanent visual loss in 10–15% [2]. Early diagnosis and prompt treatment with corticosteroids have shown to be essential to protect against visual loss, which usually occurs within the first six days after symptom onset [3]. Hence, early diagnosis and initiation of treatment emerge as crucial factors in the initial management of GCA patients.

**Fig 1:**
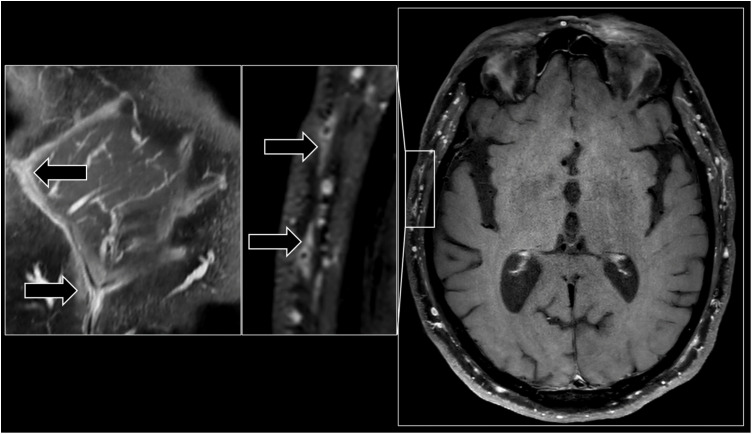
High-resolution vessel-wall MRI of temporal arteries in a patient with biopsy-proven giant cell arteritis. Seventy-two-year-old man with a positive diagnosis of GCA on post-contrast three-dimensional fat-saturated turbo spin echo high resolution vessel-wall imaging dedicated for visualizing the extracranial artery wall. Multiplanar reconstruction (left images) and axial plane (right image) showing a marked wall thickening (> 0.7 mm) and strong mural enhancement with perivascular inflammatory infiltration of temporal arteries (arrows).

Temporal artery biopsy (TAB) has long been considered the gold standard for GCA diagnosis, but the waiting time for this procedure, and for obtaining the results, can be lengthy. Vascular wall imaging, including ultrasound imaging (US) and magnetic resonance imaging (MRI), has demonstrated comparable diagnostic value if assessors are proficient in these techniques, and may now replace TAB for the diagnosis of GCA according to the 2022 American College of Rheumatology/ European League Against Rheumatism (ACR/EULAR) recommendations for the management of large vessel vasculitis [4] (Fig 1). Imaging has the advantage of being non-invasive and generally more accessible, facilitating early confirmation of the diagnosis. However, uncertainty surrounding the diagnosis in ambiguous cases, non-standardized pathways of patient management along with concerns about high-dose corticosteroid treatment in older patients can result in unnecessary hospital admissions and increased inpatient care expenses. To address these challenges, the fast-track approach has been successfully implemented in various medical fields, demonstrating reductions in mortality, morbidity, and inpatient days of care [5,6]. Rapid initiation of treatment through fast-track outpatient GCA clinic has been shown to improve visual outcomes in previous studies [7–9]. However, the cost-effectiveness of this approach has not been extensively studied.

The objective of this study was to assess the medico-economic impact of a fast-track pathway in the diagnosis and treatment of GCA patients.

## Methods

### Study design and ethics

This retrospective longitudinal study was performed in a tertiary referral center specializing in head-and-neck, ophthalmological and neurological diseases. This study was approved by our Institutional Review Board and adhered to the tenets of the Declaration of Helsinki (project ref. CE_20180925_11_TSE). Written informed consent was obtained from all subjects to access their medical data. The data were extracted and anonymized on December 1, 2023. This study follows the Strengthening the Reporting of Observational Studies in Epidemiology STROBE guideline [10].

### Population

Patients eligible for the study were identified by medical health records query registered between November 2016 and December 2022 and met the following inclusion criteria: (i) Age ≥ 50 years; (ii) new-onset diagnosis of GCA established by the presence of at least three of four clinic-biological criteria of the American College of Rheumatology, and a positive TAB or a positive vessel wall imaging among MRI, US or Positron Emission Tomography with Computed Tomography (PET-CT), following the EULAR guidelines [11].

### Fast-track pathway

The fast-track pathway was implemented in November 2018 with three main objectives: (1) to facilitate the referral of patients with suspected giant cell arteritis (GCA) to our specialized center; (2) to reduce the time to diagnosis and management of both complicated and uncomplicated GCA cases; (3) to improve the visual prognosis of patients with GCA. A specific protocolized care pathway was established based on the presence or absence of visual complications. In cases of visual complications, patients were urgently administered intravenous corticosteroid boluses (250–1000 mg/day methylprednisolone for 3 days) followed by oral corticosteroids at 1 mg/kg/day, underwent immediate confirmatory imaging for diagnosis, available 24/7, and received specialized hospital care. In the absence of visual complications, patients were scheduled for an internal medicine consultation within 24–72 hours and a day hospitalization within seven days for evaluation and initiation of treatment. A dedicated internal medicine specialist was made available 24/7. Access to confirmatory MRI imaging was facilitated. In cases of negative MRI but high clinical suspicion of GCA, additional multimodal imaging was performed, including Doppler ultrasound and/or 18F-FDG PET-CT depending on clinical presentation and availability. When diagnostic uncertainty persisted despite these investigations, a temporal artery biopsy was performed. Training sessions were conducted for emergency department junior and senior doctors, emergency reception nurses, ophthalmologists and private physicians, through a free e-learning module developed by an internal medicine specialist (“BLINDED”). A Twitter account (“BLINDED”) was also created to raise awareness among physicians and the public about GCA diagnosis.

### Data collection

The following demographic, medical and medico-economic data were retrieved through medical health records query: age, sex, current treatment, cardiovascular risk factors, medical and surgical history, clinical signs, ACR diagnostic criteria, presence and type of visual impairment, ophthalmological examination, date of onset of symptoms, date of first consultation, date of imaging, date of start of treatment. Afferent visual complications were defined as ischemic damage to the optic nerve or retina (including arteritic anterior ischemic optic neuropathy, central or branch retinal artery occlusion, cilioretinal artery occlusion, or paracentral acute middle maculopathy). Efferent visual complications were defined as oculomotor abnormalities resulting from ischemia of the extraocular muscles or cranial nerves III, IV, or VI, leading to diplopia or ocular motility restriction. The overall cost of care was calculated on the basis of diagnostic tests required, length of hospital stay and treatment administered.

These data were compared between groups of patients managed before and those managed after the implementation of the fast-track pathway in November 2018.

### Statistical analyses

Statistical analyses were conducted by a statistician (“BLINDED”) on R software (v4.0.3) [12]. Categorical variables are given as count (percentage), continuous variables as median (interquartile range) or mean (± standard deviation). For comparisons between groups, the independent samples t-test for continuous variables and Chi-2 test or Fisher’s exact test (where appropriate) for categorical variables were applied. For non-parametric numerical variables, the Wilcoxon signed-rank test was applied. The level of statistical significance was set at P < 0.05 after Bonferroni correction for multiple testing.

## Results

### Population

A total of 135 patients (mean age, 76 years ± 8, range 59–94 years, 82/135 (61%) women) were enrolled, including 23 patients before and 112 patients after the FTP implementation. Baseline demographic and clinical data of the two groups were similar. Most patients had a cephalic presentation (133/135, 99%), and 75/135 patients (56%) had either afferent or efferent ophthalmologic symptoms at diagnosis (Table 1).

**Table 1:** Population demographic and clinic-biological data.

	Before FTP implementation	After FTP implementation	Overall	*P*-value
	N = 23	N = 112	N = 135	
**Age (years)**	77 ± 8 [59–94]	76 ± 8 [59–94]	76 ± 8 [59–94]	0.67
**Sex (female)**	12 (52%)	70 (62%)	82 (61%)	0.36
**Cardiovascular risk factors**				
Diabetes	5 (22%)	13 (12%)	28 (21%)	0.20
Hypertension	12 (55%)	55 (49%)	67 (50%)	0.82
Smoking	3 (13%)	16 (14%)	19 (14%)	0.68
**Treatment before diagnosis**				
** **Platelet antiaggregant	9 (39%)	29 (26%)	38 (28%)	0.20
** **Anticoagulant	0 (0%)	12 (11%)	12 (9%)	0.10
Statin	5 (22%)	31 (28%)	36 (27%)	0.56
ACE inhibitors or ARB	8 (35%)	39 (35%)	47 (35%)	1
**CHADS2VASC score**	3 ± 1 [0–7]	3 ± 1 [0–7]	3 ± 1 [0–7]	1
**Clinical data**				
Cephalic presentation	23 (100%)	110 (98%)	133 (99%)	0.52
Headache	20 (87%)	84 (75%)	104 (77%)	0.21
Jaw claudication	14 (61%)	58 (52%)	72 (53%)	0.43
Scalp tenderness	14 (61%)	58 (52%)	72 (53%)	0.51
Aorto-arteritic presentation	4 (17%)	10 (10%)	14 (11%)	0.31
Polymyalgia rheumatica	8 (35%)	23 (21%)	31 (23%)	0.15
Neurological symptoms	3 (13%)	12 (11%)	15 (11%)	0.78
Afferent visual complication	12 (52%)	56 (50%)	68 (50%)	0.85
- A-AION	9 (39%)	42 (38%)	51 (38%)	
- CRAO	5 (22%)	11 (10%)	16 (12%)	
- Other	1 (4%)*	11 (10%)**	12 (9%)	
Efferent visual complication	3 (13%)	24 (22%)	27 (20%)	0.34
**C-reactive protein (mg/l)**	57 ± 43	53 ± 49	54 ± 48	0.73
**Erythrocyte sedimentation rate (ESR)**	70 ± 40	67 ± 31	67 ± 32	0.7

ACE: Angiotensin converting enzyme; ARB: angiotensin receptor blocker; FTP: Fast-track pathway; A-AION: arteritic anterior ischemic optic neuropathy; CRAO: central retinal artery occlusion; * 1 branch retinal artery occlusion; ** 6 cilioretinal occlusion, 3 paracentral acute middle maculopathy, 2 non-arteritic ischemic optic neuropathy.

### Hospitalization pattern

After FTP implementation, fewer patients were treated in full hospitalization (69/112 (62%) vs 22/23 (96%), P < 0.01). More patients were treated in the setting of day hospitalization (23/112 (21%) vs 0/23 (0%), P < 0.01) or outpatient care (20/112 (18%) vs 1/23 (4%), P < 0.01). Hospitalizations were shorter after implementation of FTP (4.9 ± 5.5 days vs 7.0 ± 5.5 days, P = 0.11).

### Treatment and visual prognosis

After FTP implementation, time from symptom onset to treatment initiation was lower, with more patients treated during the first month after symptom onset (58/112 (54%) vs 5/23 (22%), P < 0.01). Cumulative intravenous corticosteroid exposure was significantly lower (1679 ± 760 mg vs 2295 ± 1055 mg before FTP, *P* = 0.02). In addition, steroid-sparing agents were prescribed less frequently after FTP: methotrexate was used in 4/23 (17%) patients before FTP versus 5/112 (4%) after FTP, and tocilizumab in 10/23 (43%) versus 22/112 (24%). While the visual acuity was not statistically different between groups at diagnosis (2 log MAR vs 2.3 log MAR, P = 0.12), final visual acuity was higher in the FTP group (2 log MAR vs 2.6 log MAR, P < 0.01), as detailed in Table 2.

**Table 2:** Patients outcome.

	Before FTP implementation	After FTP implementation	Overall	*P*-value
	N = 23	N = 112	N = 135	
**Ophthalmologic symptoms**	13 (56%)	62 (55%)	75 (56%)	0.92
Bilateral eye involvement at diagnosis	1 (4%)	15 (13%)	16 (12%)	0.17
Bilateralization under treatment	3/12 (25%)	4/47 (9%)	7/59 (12%)	0.11
Bilateral eye involvement after treatment	4 (17%)	19 (17%)	23 (17%)	0.95
Visual acuity at diagnosis (log MAR)	2.1 ± 1.1 [0.0 – 3.0]	1.8 ± 0.8 [0.0 – 2.6]	1.8 ± 0.9 [0.0 – 3.0]	0.13
Visual acuity after treatment (log MAR)	2.3 ± 0.8 [0.0 – 3.0]	1.6 ± 0.8 [0.0 – 2.6]	1.8 ± 0.9 [0.0 – 3.0]	**< 0.01**
**Treatment**				
Intravenous CS bolus	11/23 (48%)	64/112 (57%)	75/135 (56%)	0.41
Intravenous CS cumulative dose (mg)	2295 ± 1055 [1240–4500]	1679 ± 760 [720 −5000]	1769 ± 831 [720–5000]	**0.02**
Methotrexate (number of patients)	4 (17%)	5 (4%)	9 (7%)	**0.02**
Tocilizumab (number of patients)	10 (43%)	22 (20%)	32 (24%)	**0.02**

CS: Corticosteroids; FTP: Fast-track pathway; MAR: minimum angle of resolution

### Temporal artery biopsy vs vascular imaging

After FTP implementation, significantly fewer patients underwent a TAB (13/112 (12%) vs 13/23 (62%), P < 0.01). More patients benefited from non-invasive vascular imaging using MRI (103/112 (94%) vs 15/23 (65%), P < 0.01), as detailed in Table 3.

**Table 3:** Medico-economic analysis: hospitalization pattern, laboratory results and imaging.

	Before FTP implementation	After FTP implementation	Overall	*P*-value
	N = 23	N = 112	N = 135	
**Hospitalization pattern**				
Full hospitalization	22 (96%)	69 (62%)	91 (67%)	**< 0.01**
Day hospital	0 (0%)	23 (21%)	23 (17%)	**< 0.01**
Outpatient care	1 (4%)	20 (18%)	21 (16%)	**< 0.01**
**Time before day hospital** (days)	NA	4 ± 3	4 ± 3	NA
**Length of hospitalization** (days)	7 ± 5 [2–18]	5 ± 5 [0–31]	6 ± 5 [0–31]	0.21
**Time from symptom onset to treatment, less than one month**	5 (22%)	58 (52%)	63 (47%)	**< 0.01**
**TAB**	21 (91%)	19 (17%)	40 (30%)	**< 0.01**
**TAB positivity**	13/21 (62%)	13/19 (68%)	26/40 (65%)	0.28
**Vascular imaging**				
MRI	15 (65%)	103 (94%)	118 (89%)	**< 0.01**
US	18 (78%)	38 (34%)	56 (41%)	**< 0.01**
PET-CT	5 (22%)	9 (8%)	14 (10%)	0.05
**Health care costs**				
Total	4486€ ± 3193	3672€ ± 2861		0.23
Hospitalization costs	4668€ ± 3023	3680€ ± 2752		0.15
Consultation and examination costs	447€ ± 368	389€ ± 266		0.39

FTP: Fast-track pathway; MRI: Magnetic resonance imaging; PET-CT: Positron emission tomography with computed tomography; SD: Standard deviation; TAB: Temporal artery biopsy; US: Ultrasound;

### Lower medical costs

The FTP decreased the average global medical costs from 4486€ ± 3193–3672€ ± 2861 (P = 0.23). Hospitalization costs decreased from 4668€ ± 3023–3680€ ± 2752, P = 0.15), and consultation/examination costs from 447€ ± 368–389€ ± 266, P = 0.39 (Table 3).

## Discussion

In this study, we showed that a fast-track pathway for the management of GCA patients may improve visual prognosis and decrease healthcare costs by encouraging outpatient or day hospitalization rather than full hospitalization. Easier access to non-invasive diagnostic modalities has reduced the need for temporal artery biopsies, enabling faster, non-invasive diagnostic confirmation. After implementation of the FTP, patients were treated earlier and their final visual acuity was better

According to scientific projections, by 2050, more than 3 million people will have been diagnosed with GCA in Europe, North America, and Oceania [13]. About 500 000 people will be visually impaired worldwide, and the projected cost of visual impairment due to GCA will exceed 76 billion dollars in the USA. The cost of the inpatient care for the GCA patients could be about 1 billion dollars, and management of steroid-related adverse events will increase costs further, with steroid-induced fractures estimated to total 6 billion dollars [13]. These projections underline the need to improve the prevention and early management of GCA, while at the same time reducing healthcare costs, particularly those associated with full hospitalization. With this in mind, FTPs appear to be an indispensable tool. The fast-track approach has been implemented in various medical fields, and its remarkable achievements in decreasing mortality, morbidity, and the length of hospital stays have been widely acknowledged [5,6]. Regarding GCA, several studies have shown the ability of FTPs to reduce permanent visual impairment, which is in line with our results [7–9]. To our knowledge, our study is the first to evaluate the medico-economic impact of such an FTP for GCA patients in Europe.

Imaging plays a pivotal role in the diagnostic workup of GCA [11,17]. MRI has emerged as a modality of choice, given its widespread availability 24/7 in our center and increasingly in other centers worldwide, allowing for rapid diagnostic confirmation or exclusion. High-resolution MRI of cranial and extracranial arteries has proven to be a valuable tool in detecting vascular inflammation, offering a non-invasive and accurate alternative to temporal artery biopsy [14,15]. This aligns with our previous work, including studies on the decision-making algorithm for GCA diagnosis, which identified MRI as the best initial diagnostic examination [16]. The use of MRI not only streamlines the diagnostic process but also reduces delays in treatment initiation, a critical factor in preventing visual symptoms and other complications [17].

Beyond diagnostic improvements, the FTP produced structural changes with clear economic implications. The transition from full hospitalization to day-hospital or outpatient management reduced costs, workload, and pressure on hospital infrastructure, while enhancing patient comfort. Although mean total healthcare costs decreased by €814 per patient, this difference did not reach statistical significance. This likely reflects sample size limitations and interindividual variability rather than an absence of true effect, suggesting instead a consistent trend toward cost reduction. Importantly, these conclusions rely on real-world billing data from the French public healthcare system, and cost patterns may differ internationally depending on organization and reimbursement models. Future multicenter or randomized studies are warranted to confirm the reproducibility and generalizability of these findings.

Earlier diagnosis under the FTP not only improved visual outcomes but also optimized therapeutic strategies. Corticosteroid therapy primarily prevents new ischemic events rather than reverses existing visual loss [1]. The better final visual acuity observed post-FTP likely reflects earlier treatment, preventing bilateral involvement or additional ischemic episodes, rather than recovery of pre-existing deficits. The slightly higher rate of bilateral involvement at diagnosis post-FTP probably results from the before/after design and small size of the pre-FTP cohort rather than a causal effect.

In parallel, FTP implementation was associated with reduced cumulative intravenous corticosteroid exposure and less frequent use of steroid-sparing agents such as methotrexate or tocilizumab. Intravenous boluses remained reserved for patients with severe ocular or neurologic involvement, but doses became more tailored over time, typically ranging from 250–500 mg/day instead of systematic 1000 mg/day regimens. This evolution reflects improved interdisciplinary coordination and enhanced practitioner awareness within the FTP framework.The FTP combined structural innovations (24/7 access to specialists, immediate imaging) with dedicated educational initiatives for all healthcare professionals involved. Given the before/after design, it is not possible to disentangle the individual impact of these components. However, the observed global improvement in visual outcomes, corticosteroid use, and hospitalization patterns likely reflects the synergistic effect of structural reorganization and increased awareness.This study has several limitations. First, as a retrospective study performed in tertiary referral center specializing in head and neck diseases, there was a selection bias towards patients with visual impairment. The high prevalence of visual impairment at diagnosis may explain the low power of the study to demonstrate the impact of FTP on the primary prevention of visual impairment. Indeed, it is important to note that the goal of corticosteroid therapy is to prevent the onset of visual symptoms or their bilateralization, but it does usually not allow for recovery of already established visual impairment [1]. Therefore, the better final visual acuity observed after FTP implementation should not be interpreted as a reversal of pre-existing visual loss, but rather as the consequence of earlier treatment preventing further ischemic events or bilateralization. This interpretation is further supported by the absence of significant difference in initial visual acuity between groups and by the lower rate of bilateralization under treatment observed after FTP implementation. Second, due to the retrospective design of the study, few data were available on the medium- and long-term follow-up of patients, and therefore on possible relapses and duration of treatment in each group. However, in the literature, the early diagnosis and treatment was not associated with higher relapse rates [3]. Another limitation is that visual function was assessed based on central visual acuity only, as systematic visual field testing was not available in all patients. Central visual acuity primarily reflects foveolar function and may not capture peripheral visual field loss. Future prospective studies with systematic follow-up will be essential to address these outcomes.

Lastly, the implementation of FTPs in our center has introduced significant changes in clinical practices, emphasizing a multidisciplinary approach involving radiologists, rheumatologists, neurologists and ophthalmologists. Educating healthcare professionals through dedicated pedagogical tools and training sessions has been essential in ensuring the effective use of the FTP. By increasing awareness among referring physicians and the medical team, we have been able to act on multiple levers to optimize patient care. This multidisciplinary collaboration underscores the importance of structured pathways in achieving consistent and sustainable improvements in the management of GCA.

## Supporting information

S1 Table Summary of comparisons between before and after fast-track pathway implementation. (DOCX)
