# Supplementary material for: Fast-track pathway for giant cell arteritis: Improved visual outcomes and reduced healthcare costs
Source: PLoS One. 2025 Dec 4;20(12):e0338397. doi: 10.1371/journal.pone.0338397 (PMC12677582; doi:10.1371/journal.pone.0338397)
Supplement: S1 Table — (DOCX) [file pone.0338397.s001.docx]

**S1 Table. Summary of comparisons between before and after fast-track pathway implementation.**

| **Parameter** | **Pre-FTP (N = 23)** | **Post-FTP (N = 112)** | **p-value** |
| --- | --- | --- | --- |
| **Final visual acuity after treatment (logMAR)** | **2.3 ± 0.8 [0–3]** | **1.6 ± 0.8 [0–2.6]** | **< 0.01** |
| **Cumulative IV corticosteroid dose (mg)** | **2295 ± 1055 (n = 23)** | **1679 ± 760 (n = 112)** | **0.02** |
| **Full hospitalization** | **22 / 23 (96 %)** | **69 / 112 (62 %)** | **< 0.01** |
| **Day-hospital care** | **0 / 23 (0 %)** | **23 / 112 (21 %)** | **< 0.01** |
| **Outpatient care** | **1 / 23 (4 %)** | **20 / 112 (18 %)** | **< 0.01** |
| **Time from symptom onset < 1 month** | **5 / 23 (22 %)** | **58 / 112 (52 %)** | **< 0.01** |
| **Temporal artery biopsy performed** | **21 / 23 (91 %)** | **19 / 112 (17 %)** | **< 0.01** |
| **MRI performed** | **15 / 23 (65 %)** | **103 / 112 (94 %)** | **< 0.01** |
| **Ultrasound performed** | **18 / 23 (78 %)** | **38 / 112 (34 %)** | **< 0.01** |
| **PET-CT performed** | **5 / 23 (22 %)** | **9 / 112 (8 %)** | **0.05** |
| **Hospitalization cost (€)** | **4668 ± 3023 (n = 23)** | **3680 ± 2752 (n = 112)** | **0.15** |
| **Total medical cost (€)** | **4486 ± 3193 (n = 23)** | **3672 ± 2861 (n = 112)** | **0.23** |
| **Consultations + tests (€)** | **447 ± 368 (n = 23)** | **389 ± 266 (n = 112)** | **0.39** |
| **C-reactive protein (mg/L)** | **57 ± 43 (n = 23)** | **53 ± 49 (n = 112)** | **0.73** |
| **Erythrocyte sedimentation rate (mm/h)** | **70 ± 40 (n = 23)** | **67 ± 31 (n = 112)** | **0.70** |
